# Resolution Requirements in Stochastic Field Simulation of Turbulent Premixed Flames

**DOI:** 10.1007/s10494-018-9953-z

**Published:** 2018-08-11

**Authors:** M. A. Picciani, E. S. Richardson, S. Navarro-Martinez

**Affiliations:** 1 Faculty of Engineering and the Environment, University of Southampton, Southampton, UK; 2 Department of Mechanical Engineering, Imperial College London, London, UK

**Keywords:** Stochastic fields, Probability density function, Premixed combustion, Resolution requirements, Turbulent combustion

## Abstract

The spatial resolution requirements of the Stochastic Fields probability density function approach are investigated in the context of turbulent premixed combustion simulation. The Stochastic Fields approach is an attractive way to implement a transported Probability Density Function modelling framework into Large Eddy Simulations of turbulent combustion. In premixed combustion LES, the numerical grid should resolve flame-like structures that arise from solution of the Stochastic Fields equation. Through analysis of Stochastic Fields simulations of a freely-propagating planar turbulent premixed flame, it is shown that the flame-like structures in the Stochastic Fields simulations can be orders of magnitude narrower than the LES filter length scale. The under-resolution is worst for low Karlovitz number combustion, where the thickness of the Stochastic Fields flame structures is on the order of the laminar flame thickness. The effect of resolution on LES predictions is then assessed by performing LES of a laboratory Bunsen flame and comparing the effect of refining the grid spacing and filter length scale independently. The usual practice of setting the LES filter length scale equal to grid spacing leads to severe under-resolution and numerical thickening of the flame, and to substantial error in the turbulent flame speed. The numerical resolution required for accurate solution of the Stochastic Fields equations is prohibitive for many practical applications involving high-pressure premixed combustion. This motivates development of a Thickened Stochastic Fields approach (Picciani et al. Flow Turbul. Combust. **X**, YYY (2018) in order to ensure the numerical accuracy of Stochastic Fields simulations.

## Introduction

Modelling of chemical reaction terms in turbulent combustion simulations is complicated by the non-linear dependence of reaction rates on fluctuations in composition and temperature. The Probability Density Function (PDF) modelling approach is attractive because, once the joint-scalar PDF for composition and temperature is known, the chemical reaction terms needed for Reynolds-Averaged Navier-Stokes (RANS) or Large Eddy Simulation (LES) appear in closed form. In principle, the joint-scalar transported PDF approach is applicable in RANS and LES across all modes of turbulent combustion—including the limiting cases of non-premixed and perfectly-premixed combustion—provided that turbulent transport and micro-mixing effects are modelled adequately and that the PDF equation is solved accurately. The present study investigates the use of the Stochastic Fields approach [1, 2] for modelling the joint-scalar PDF evolution, and considers the requirements for obtaining numerically accurate solutions in the challenging case of turbulent premixed combustion.

The Stochastic Fields formulation has been developed rigorously through independent contributions by Valiño [1, 2] and Sabel’nikov [3]. The one-point probability density function of composition throughout a spatial solution domain is modelled by an ensemble of composition fields that also span the spatial solution domain. Each of the composition fields evolves according to a stochastic differential equation given in modelled form by Valiño et al. [2] as,

1 $$\begin{array}{@{}rcl@{}} \overline{\rho}\mathrm{d}\boldsymbol{\zeta}_{(i)}&=&-\overline{\rho}\widetilde{\mathbf{u}}\cdot\nabla{\boldsymbol{\zeta}_{(i)}}\mathrm{d}t+\nabla\left( \overline{\rho} (D+D_{T})\nabla{\boldsymbol{\zeta}_{(i)}}\right)\mathrm{d}t +\overline{\rho}\sqrt{2D_{T}}\nabla\boldsymbol{\zeta}_{(i)}d\mathbf{W}_{(i)}\\ &&-\frac{\overline{\rho}}{\tau_{T}}\left( \boldsymbol{\zeta}_{(i)}-\widetilde{\boldsymbol{\zeta}}\right)\mathrm{d}t+\overline{\rho}\dot{\boldsymbol{\omega}}(\boldsymbol{\zeta}_{(i)})\mathrm{d}t. \end{array} $$

where ***ζ***_(*i*)_(**x**, *t*) is the composition vector on the *i* th field. The terms on the right hand side represent the evolution of the stochastic field composition due to advection by the mean (or resolved) velocity; spatial diffusion by molecular *D*, and turbulent *D*_*T*_, diffusivities; turbulent advection of fields relative to one another as modelled by a Wiener process where *d***W** is a normally-distributed Markovian random increment with zero mean and variance equal to the time step *dt*; the unresolved scalar dissipation processes modelled by interaction by exchange with the mean (IEM) [4] with dissipation time scale *τ*_*T*_; and the vector of chemical reaction source terms $\boldsymbol {\dot {\omega }}(\boldsymbol {\zeta }_{(i)})$.

The Stochastic Fields approach is an attractive way to implement transported probability density function modelling for turbulent reacting flows for two main reasons: It guarantees a density field that is continuous and differentiable in space, and it can exploit Eulerian solution methods and domain decomposition schemes similar to those implemented for the momentum equations. Following studies of the Stochastic Fields approach in a range of non-premixed combustion scenarios including Refs. [5, 6], a smaller number of Stochastic Fields simulations of premixed combustion have been reported [7–9]. The degree of predictive accuracy shown in the case of premixed combustion is encouraging because the sub-models employed, such as the interaction by exchange with the mean (IEM) micro-mixing model [4], are essentially unchanged from those employed in previous non-premixed modelling efforts. Investigations of Lagrangian particle-based implementations of transported PDF modelling in flamelet combustion regimes suggest that turbulent flame predictions can be improved by applying alternative micro-mixing models that enforce localness [10–12] and also account for preferential molecular transport [13]. However, such micro-mixing models have not yet been adapted for use with the Stochastic Fields approach and are beyond the scope of the current work.

Given that reaction fronts found in premixed combustion have inherent length scales that may be smaller than the Kolmogorov scale characterizing the smallest structures of the turbulent flow [14]. It is not evident a priori from Eq. 1 what spatial resolution is required in order to obtain an accurate solution of the Stochastic Fields in turbulent premixed combustion. Therefore, the objective of this study is to investigate the spatial resolution requirements of the Stochastic Fields equations in turbulent premixed combustion applications. In particular, it is necessary to assess whether the usual practice in LES of setting the filter length scale equal to the grid spacing might lead to significant errors. In the following sections, resolution requirements are discussed in the context of premixed combustion, then the resolution requirements are investigated numerically in the subsequent sections, first in a simplified one-dimensional test problem, and then with respect to an LES of a premixed piloted stoichiometric Bunsen flame experiment by Chen et al. [15].

## Numerical Resolution of the Stochastic Fields Equation in Premixed Combustion

Spatial resolution requirements of the Stochastic Fields equations in LES of premixed combustion have not been discussed in the academic literature on Stochastic Fields. In a turbulent premixed flame, each Stochastic Field contains a reaction front that resembles a premixed flame. Strictly, the reaction fronts of the Stochastic Fields are not required to correspond to actual flame fronts in a physical flow, and therefore the term “reaction front” is used to avoid confusion. In turbulent premixed combustion, the averaged thickness of the reaction front across all individual Stochastic Fields is necessarily less than the thickness of the ensemble average of all the stochastic fields.

The combined effect of an exchange with the mean micro-mixing model and an eddy-diffusivity model for the unresolved turbulent transport is to thicken the individual Stochastic Fields relative to the average thickness of the instantaneous flame fronts that would be expected in the actual turbulent flame. In LES, the average thickness of the individual Stochastic Field reaction fronts is expected to depend on the effective filter length scale and combustion regime. It is expected that the thermal thickness of the Stochastic Fields reaction front is somewhere between the thermal thickness of an unstrained laminar premixed flame and the thermal thickness of the ensemble-averaged (RANS) or resolved (LES) temperature field.

The influence of the combustion regime on Stochastic Field thicknesses and resolution is such: Karlovitz numbers much greater than unity (i.e. broken reaction zones) have the effect of thickening instantaneous flame fronts [14], and therefore also thicken the Stochastic Field reaction fronts, reducing resolution requirements. Conversely, the most stringent resolution requirements are expected for low Karlovitz number flames that characterise combustion in the flamelet regime. Flamelet-type combustion is prevalent in most important premixed combustion applications including spark-ignition engines and industrial gas turbines. However, it is not evident from a priori analysis of Eq. 1 whether a given grid spacing will be sufficient to resolve the individual Stochastic Fields, and resolution requirements need to be investigated numerically.

The grid spacing required in order to numerically resolve a reaction-diffusion front varies depending on the numerical discretisation employed, the details of the chemistry and transport models employed, and the numerical accuracy desired. For high-accuracy simulation of premixed combustion with a detailed chemistry model, more than twenty points may be required within each reaction-diffusion front. Whereas five points within a reaction-diffusion front may be taken as an absolute minimum requirement for less accurate engineering simulations with simple (e.g. single-step) chemistry models [16]. Low-order numerical methods and TVD schemes used for some LES of turbulent reacting flows can remain stable even when the governing equations are under resolved, providing numerical diffusion that spreads the reaction fronts across multiple grid points.

In implicitly-filtered LES, the effective filter length scale used in modelling for the unclosed sub-filter scale terms usually depends on the grid spacing. Previous Stochastic Fields LES studies [5–9] have set the filter length scale equal to the grid spacing (or the cube root of the cell volume). When the filter length scale depends on the grid spacing, the effects of numerical resolution errors cannot be distinguished from scale-dependence of the sub-grid modelling. Vreman et al. [17] investigated the effects of numerical error in non-reacting LES by changing the filter scale independently from the grid spacing. For a fixed filter scale they found significant differences in LES predictions between simulations with the grid spacing equal to either the filter scale or one half of the filter scale. They did not proceed to refine the grid to the extent that grid-independence of the predictions was demonstrated, presumably due to the computational expense of further refining the three-dimensional grid. The approach of refining the numerical resolution while keeping the filter length scale unchanged is also appropriate for assessment of numerical error and resolution requirements in the Stochastic Fields equations and this approach is employed in this study. Due to the computational expense of highly-resolved three-dimensional Stochastic Fields simulations, the numerical resolution requirements are assessed across a wide range of combustion regimes using in a simplified one-dimensional test case relating to freely-propagating turbulent flames, before verifying the conclusions from the one-dimensional study in a three-dimensional LES of a turbulent premixed Bunsen flame [15].

## Formulation

**Fig. 1:**
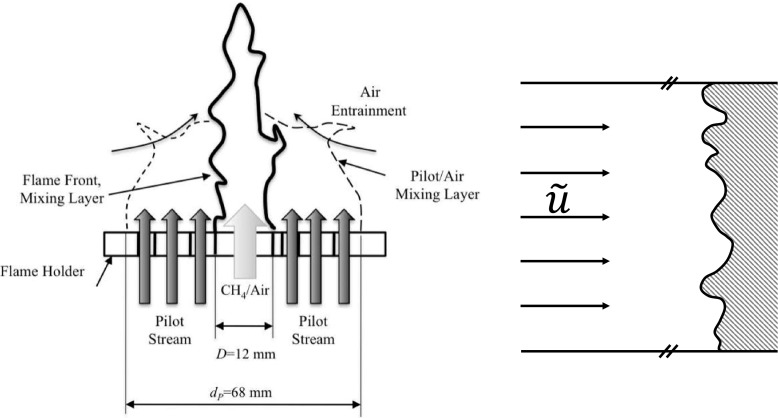
Schematic diagrams of the turbulent premixed Bunsen flame case (left) and of the freely-propagating planar turbulent premixed flame test case (right)

The statistically-stationary freely-propagating planar turbulent premixed flame test case and the turbulent premixed Bunsen flame of Chen et al. [15] are illustrated schematically in Fig. 1.

### Freely-propagating turbulent flame

#### RANS closure

The planar turbulent premixed flame is statistically homogeneous parallel to the flame and it is investigated initially using a one-dimensional Reynolds Averaged Stochastic Fields simulation. The turbulence is assumed to be uniform throughout the domain and across the flame, and specified by setting the ratio of the rms velocity fluctuation to the laminar flame speed *u*′/*S*_*L*_, and the ratio of the integral length scale of the turbulence normalised by the thermal thickness of the laminar flame, *L*_*T*_/*δ*_*L*_. The turbulent diffusivity and mixing time scale required in Eq. 1 are then modelled by the following relations:

2 $$ D_{T}=C_{\mu}u^{\prime}L_{T}, $$

and

3 $$ \tau_{T}=\frac{C_{\phi}L_{T}}{2u^{\prime}}. $$

in which *C*_*μ*_ = 0.09 [18] and *C*_*ϕ*_ = 2.0 (*μ*_*L*_/*μ*_*T*_ + 1), where the factor (*μ*_*L*_/*μ*_*T*_ + 1) models behaviour at low Reynolds numbers [19].

#### LES closure

Large Eddy Simulation is inherently three-dimensional. However the resolved flame front in LES of premixed turbulent combustion is typically thin with respect to the radius of curvature of the resolved flame front, across much of the flame surface area. Therefore, over much of the flame area, it is valid to approximate the molecular and sub-filter scale turbulent transport within the resolved flame front as one-dimensional in the direction perpendicular to the resolved flame front.

In order to investigate resolution requirements in the LES context, one-dimensional Stochastic Fields simulations are performed using sub-models for the sub-filter scale turbulent diffusivity and dissipation time scale that depend on a notional LES filter length scale Δ and the corresponding sub-filter scale velocity fluctuation $u_{{\Delta }}^{\prime }$. The one-dimensional simulations may be interpreted loosely as representing the transport along a line passing perpendicularly through a LES-resolved flame front, assuming that the propagation of the LES-resolved flame front is quasi-steady and unaffected by other flame fronts, by curvature, or by resolved strain (except to the extent that the resolved strain results in generation of sub-filter scale velocity fluctuations characterised by $u_{{\Delta }}^{\prime }$). The sub-filter scale diffusivity and dissipation time scales in Eq. 1 are then modelled by Eqs. 2 and 3, replacing the turbulence length scale *L*_*T*_ with the filter scale Δ, and the turbulent velocity *u*′ with the sub-filter scale velocity $u^{\prime }_{{\Delta }}$, and setting coefficients *C*_*μ*_ = 0.09 and *C*_*ϕ*_ = 2.0 (*μ*_*L*_/*μ*_*T*_ + 1) as in the RANS case.

In both the RANS and LES context, the turbulent premixed combustion regime is characterised by the Karlovitz number, Ka. In terms of integral length scale, the Karlovitz number can be approximated by

4 $$ \text{Ka}=\left[\left( \frac{u^{\prime}}{S_{L}}\right)^{3}\frac{\delta_{L}}{L_{T}}\right]^{1/2} $$

which can be obtained though conventional scaling analysis. In the LES context, for filter length scales in the inertial sub-range of the turbulence spectrum, the filter scale Karlovitz number is given by,

5 $$ \text{Ka}_{{\Delta}}=\left[\left( \frac{u_{{\Delta}}^{\prime}}{S_{L}}\right)^{3}\frac{\delta_{L}}{{\Delta}}\right]^{1/2}. $$

With the assumption of constant dissipation in the inertial range, the Karlovitz number is scale invariant [16] and equal to the integral scale Karlovitz number, Ka_Δ_ = Ka. Since LES relies on selection of a filter length scale in the inertial sub-range, the effect of choosing different ratios of the filter length scale to laminar flame thickness (Δ/*δ*_*L*_) for simulation of a particular turbulent flame regime can be investigated by fixing Karlovitz number and evaluating the corresponding sub-filter scale velocity fluctuation as,

6 $$ u_{{\Delta}}^{\prime}=S_{L}\text{Ka}_{{\Delta}}^{2/3}\left( \frac{{\Delta}}{\delta_{L}}\right)^{1/3}. $$

The RANS model described previously is recovered in the limit that Δ → *L*_*T*_, giving $u_{{\Delta }}^{\prime }\to u^{\prime }$.

In the following one-dimensional study, combinations of Karlovitz numbers, Ka ∈ [0.5, 1, 5, 10, 20, 30, 40, 50], and three characteristic, normalised length scales *L*_*T*_/*δ*_*L*_ of [1, 2.5, 5.0] are used for the one-dimensional simulations. These parameters span a range of premixed combustion regimes that would be typical of currently advanced LES of spark ignition and industrial gas turbines using between 10 and 100 million cells, as well as the conditions in the three-dimensional LES test case studied here.

### Turbulent Bunsen flame configuration

The F3 turbulent premixed Bunsen flame described by Chen et al. [15] is simulated using Stochastic Fields-LES. The flame is characterised by Karlovitz numbers of order unity, indicating that combustion takes place across the flamelet and thin reaction zone regimes. The flame has relatively simple boundary conditions and has served as the basis for numerous investigations of PDF modelling for turbulent premixed combustion [7, 10, 20–22].

A 12 mm diameter nozzle delivers a turbulent jet of 300 K stoichiometric methane-air with bulk velocity 30 ms^− 1^. The flame is stabilised by a ring of stoichiometric methane-air pilot flames surrounding the nozzle with 68 mm outer diameter and bulk velocity 1.32 ms^− 1^. Further downstream the flame entrains 300 K air at 1 atm from a quiescent laboratory environment. Profiles of mean and rms velocity fluctuations are reported in [15] and are used to set the numerical inflow profiles for the simulation.

The largest source of uncertainty with this particular test case is the pilot temperature due to heat loss between the pilot flame, the burner, and the environment. Various numerical simulations of this series of flames have used different values of the pilot flame temperature, however in this work, it has been estimated as in previous LES studies [7] as 1785 K. Due to the use of simplified chemistry and the omission of certain chemical species, the pilot composition was modified from that outlined in [15], and is given in Table 1.

**Table 1:** Pilot Stream Composition

Species	Chen et al. [15]	Current Work
$Y_{O_{2}}$	5.00E-4	5.00E-4
$Y_{H_{2}O}$	0.1236	0.1236
$Y_{CO_{2}}$	0.15	0.15
*Y*_*CO*_	7.800E-4	–
$Y_{H_{2}}$	3.00E-5	–
*Y*_*OH*_	1.20E-4	–
$Y_{N_{2}}$	0.7247	0.7259

Contrary to the one-dimensional simulations, the spatially-filtered continuity and momentum equations [7] are closed with the constant-coefficient Smagorinsky model for the sub-filter scale turbulent stresses [23], with Smagorinsky constant equal to 0.09. The turbulent diffusivity required in Eq. 1 is modelled assuming turbulent Schmidt number equal to 0.7 and the dissipation time scale in Eq. 1 is modelled by 1/*τ*_*T*_ = *C*_*ϕ*Δ_(*D* + *D*_*T*_)/Δ^2^, with model coefficient *C*_*ϕ*_ once again scaled as proposed in [19].

### Thermo-chemical models

The premixed combustion kinetics are modelled using a one-step reaction model for methane-air flames,

7 $$ CH_{4}+ 2O_{2}\rightarrow CO_{2}+ 2H_{2}O. $$

The fuel reaction rate is modelled by the Arrhenius law,

8 $$ \dot{\omega}_{CH_{4}}=A\cdot\left( \frac{\rho Y_{CH_{4}}}{M_{CH_{4}}} \right)^{n_{CH_{4}}}\left( \frac{\rho Y_{O_{2}}}{M_{O_{2}}} \right)^{n_{O_{2}}}\exp\left( -\frac{E_{a}}{RT} \right), $$

where *T*, $Y_{CH_{4}}$, $Y_{O_{2}}$, $M_{CH_{4}}$, $M_{O_{2}}$ and *R* denote temperature, fuel and oxygen mass fractions, corresponding molar weights and the universal gas constant, respectively. The pre-exponential factor, the activation energy and the model exponents are *A* = 1.1 × 10^10^ (cgs), *E*_*a*_ = 20, 000 cal/mol, $n_{CH_{4}}= 1.0$ and $n_{O_{2}}= 0.5$. The use of such simple chemical modelling is justified by the focus of the present study on evaluation of the numerical resolution requirements of the Stochastic Fields approach, rather than assessing the absolute physical accuracy of the Stochastic Fields approach.

Temperature-dependent thermodynamic properties are modelled with NASA polynomials and, due to the inherent unity Lewis number assumption in the Stochastic Fields formulation, the Schmidt and Prandtl numbers are both set equal to 0.7, while the mixture kinematic viscosity is modelled with Wilkes law. These assumptions lead to a laminar flame speed *S*_*L*_ = 0.38 ms^− 1^, a thermal thickness of 0.408 mm and a burnt gas adiabatic temperature *T*_*b*_ = 2328 K in atmospheric stoichiometric conditions.

### Numerical implementation and simulation setup

The Stochastic Fields equation is implemented within the block-structured BOFFIN computational fluid dynamics code [7, 24]. The code is a second order accurate finite volume method based on fully implicit low-Mach-number formulation using a staggered storage arrangement. For the momentum equation convection term, an energy conserving discritization scheme is used and all other spatial derivatives are approximated by standard second order central differences. A TVD scheme is used for the convection terms in the scalar conservation equations. The stochastic field equations are solved using a weak first order temporal approximation with accuracy $\mathcal {O}\left (\sqrt {{\Delta }{t}}\right )$ based on the Euler-Maruyama scheme [25]. The Wiener process is approximated by time-step increments $dt^{1/2}{\eta ^{n}_{i}}$ where ${\eta ^{n}_{i}}$ is a {− 1, 1} dichotomic random vector [3]. The chemical source terms are solved using a Newton method-based stiff solver.

For the one-dimensional simulations, the domain was selected to be sufficiently large to ensure zero-gradient conditions at both the inflow and outflow planes. The inflow velocity is controlled in order to keep the flame brush position stationary in the domain. The grid spacing is uniform and set according to the resolution requirements of each test case. It is observed that using an equispaced numerical grid with at least 16 grid points within the average stochastic field thickness provides a grid-independent “well-resolved” Stochastic Fields solution. For the one-dimensional case 512 Stochastic Fields were used, which is sufficient to make the results presented independent of the number of stochastic fields.

The turbulent Bunsen flame is simulated using two different computational grids: a fine grid characterised by 0.5 mm grid spacing at the inlet, and a coarse grid characterised by 1.0 mm grid spacing at the inlet. Both grids are Cartesian with a region of uniform transverse grid spacing around the inlet with transverse extent equal to twice the nozzle diameter. The axial grid spacing increases linearly in the axial direction. Sixteen stochastic fields are used in the three-dimensional simulations. The computational time step for the respective cases are 4.6 *μ*s and 2.3 *μ*s. The turbulent inflow is modelled with the digital filter based method of Klein et al. [26] using the mean and rms velocity profiles from [15].

## Results and Discussion

**Fig. 2:**
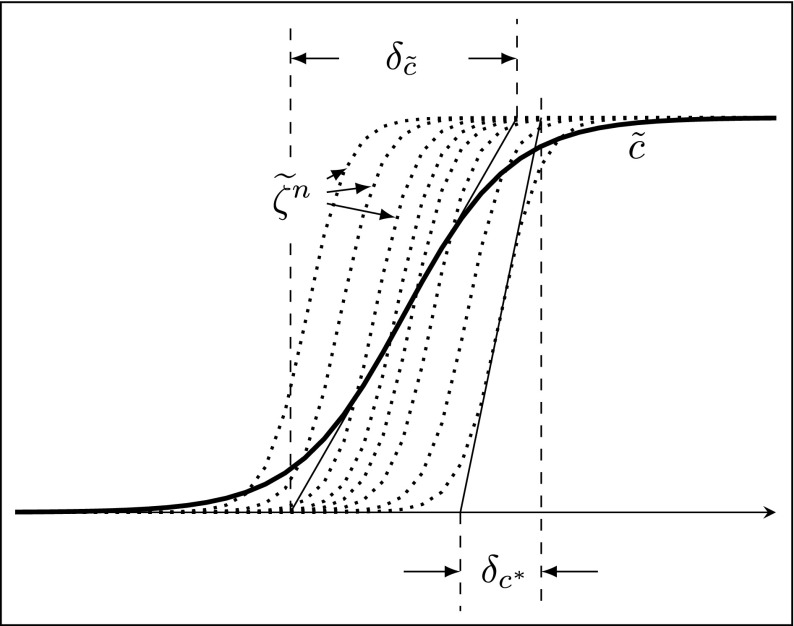
Representative diagram illustrating the definition of the thickness of the individual stochastic progress variable field, $\delta _{c^{*}}$

In order to evaluate the resolution requirements of the Stochastic Fields equation, the thermal thicknesses of the reaction fronts $\delta _{c}^{*}$ on the individual stochastic fields are evaluated as indicated in Fig. 2, ensemble averaged according to

9 $$ \left\langle\delta_{c^{*}}\right\rangle=\frac{1}{N_{s}}\sum\limits_{i = 1}^{N_{s}}\frac{1}{|\nabla{\zeta_{(i)}}|_{max}}, $$

where *N*_*s*_ is the number of stochastic fields, and then time averaged. $\langle \delta _{c^{*}}\rangle $ is referred to as the “average stochastic field thickness” and should not be confused with the mean/filtered flame thickness ($\delta _{\tilde {c}}$) which is the thickness based on the maximum gradient of the ensemble averaged progress variable field.

Additionally, the impact of resolution on the mean/filtered flame speed was examined. These flame speeds were calculated based on the consumption rate as

10 $$ S_{T}=\frac{1}{\rho_{u}Y_{f}A}{\int}_{V}\frac{1}{N_{s}}\sum\limits_{i = 1}^{N_{s}}\dot{\omega}_{(i)}\text{dV}, $$

where *ρ*_*u*_ is the unburnt gas density, *Y*_*f*_, the mass fraction of fuel in the premixed reactants, *A*, the domain cross sectional area, and $\dot {\omega }_{(i)}$, the instantaneous reaction rate on field *i*. The instantaneous consumption speeds were also time averaged.

### Freely-propagating turbulent flame RANS

**Fig. 3:**
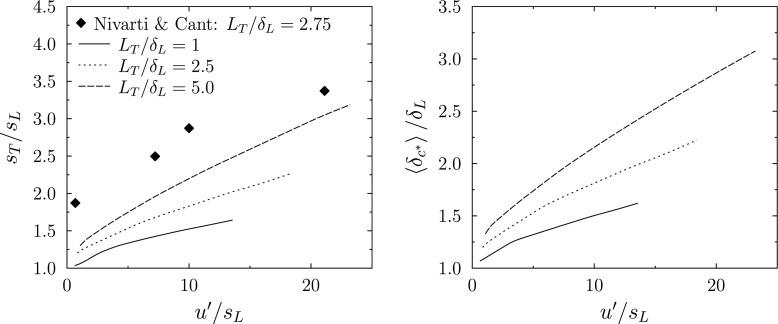
RANS Stochastic Fields results for freely-propagating time averaged turbulent flame speed (left) and for time averaged average thermal thickness of the stochastic fields (right) versus $u^{\prime }/S_{L}$ for *L*_*T*_/*δ*_*L*_ = 1, 2.5, 5, and experimental results from Nivarti and Cant [27]

The one-dimensional RANS model is applied to the freely-propagating turbulent premixed flame for a range of *u*′/*S*_*L*_ and *L*_*T*_/*δ*_*L*_, and the turbulent flame speed and stochastic field thickness predictions are reported in Fig. 3. Since one objective is to apply the Stochastic Fields in the LES context, in which the sub-filter Reynolds number is typically small, the predictions are shown for values of *L*_*t*_/*δ*_*L*_ up to five, corresponding to relatively low Reynolds number. The predictions are presented alongside DNS flame speed data for freely propagating stoichiometric methane-air flames by Nivarti and Cant [27] with similar *L*_*T*_/*δ*_*L*_, and based on a one-step chemical reaction model similar to that used in the Stochastic Fields simulations.

Previous experimental turbulent flame speed measurements [28] and also the low-Reynolds number Nivarti and Cant DNS data in Fig. 3 show a near-linear increase of *S*_*T*_/*S*_*L*_ at low *u*′/*S*_*L*_, followed by a bending effect. The bending effect is attributed to the volume occupied by the turbulent flame becoming saturated with flame surface area. Previous experiments [28] indicate that the bending occurs earlier at lower Reynolds number (smaller *L*_*T*_/*δ*_*L*_). Theory [14] and experimental observations [28] indicate that, for thermo-diffusively-stable flames, the turbulent flame speed tends towards the planar laminar flame speed (*S*_*T*_/*S*_*L*_ → 1) in the limit *u*′/*S*_*L*_ → 0. In contrast, the DNS data of Nivarti and Cant [27] show an unexpected value of *S*_*T*_/*S*_*L*_ = 1.75 at *u*′/*S*_*L*_ → 0.

The Stochastic Fields predictions for *S*_*T*_/*S*_*L*_ in Fig. 3 show a similar trend to the Nivarti and Cant [27] DNS data, including near-linear increase at low *u*′/*S*_*L*_ and a gradual bending effect over the range of *u*′/*S*_*L*_ reported. The Nivarti and Cant flame speed data (with *L*_*T*_/*δ*_*L*_ = 2.75) are consistently a factor of 1.75 greater than the Stochastic Field predictions for *L*_*T*_/*δ*_*L*_ = 2.5. Since the DNS flame speeds are higher than theoretically-expected values by the same factor, the Stochastic Fields model appears to provide satisfactory flame speed predictions for this range of conditions. The Stochastic Fields predictions are also subject to the uncertainties of modelling the unresolved diffusive and turbulent convective fluxes. The use of non-local micromixing models, such as the interaction by exchange with the mean model used here, are known to be inaccurate in Lagrangian particle RANS-PDF modelling of premixed flames [20]. However, in the Stochastic Fields approach, the simple exchange with the mean and gradient transport approaches adopted appear sufficient to produce reasonable predictions for the initial gradient and bending of *S*_*T*_/*S*_*L*_ with respect to *u*′/*S*_*L*_.

The reasons for the relative success of non-local mixing models in the context of the Stochastic Fields PDF implementation (compared to particle-PDF implementations) in premixed combustion applications may be first that the stochastic fields are continuous in space and second, that a portion of the scalar dissipation processes are accounted for by the spatial diffusion term in the Stochastic Fields equation. The coupling between the spatial diffusion and reaction terms then drives the compositions in the Stochastic Fields solutions towards a flame-like composition manifold across the reaction front, even if the micromixing model lacks the localness property and might otherwise draw the composition into regions of composition space that are in practice chemically-unrealisable.

The average thickness of the RANS Stochastic Fields presented in Fig. 3 shows that the thickness reduces towards the laminar flame thickness at low *u*′/*S*_*L*_. The thickness remains on the order of the laminar flame thickness across a wide range of turbulence conditions. Since RANS simulations would otherwise resolve only the large scale flow features that drive the integral scale turbulence (i.e. length scales greater than *L*_*T*_), the Stochastic Fields approach, requiring resolution at scales close to the much smaller laminar flame thickness, increases the resolution requirements dramatically when applied to premixed combustion.

### Freely-propagating turbulent flame LES

The one-dimensional turbulent flame simulations are analysed next in the context of LES. In particular, we examine the effect of using grid spacing equal to the filter scale Δ_*x*_ = Δ, compared to the “well-resolved” limit Δ_*x*_ ≪ Δ. The simulation case with Δ_*x*_ = Δ are described as “coarse”. All cases are simulated with 512 stochastic fields to ensure statistical convergence.

**Fig. 4:**
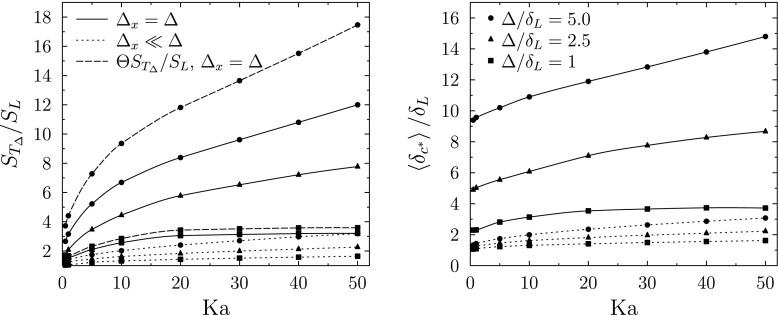
One-dimensional LES Stochastic Fields results for freely-propagating turbulent flame. Normalised resolved flame front speed (left) and normalised average stochastic field thickness (right) versus *Ka* for Δ/*δ*_*L*_ = 1, 2.5, 5. Legends apply to both sub-figures, Θ is defined in Eq. 11

Figure 4 shows that use of the coarse grid leads to a substantial error in the propagation speed of the LES-resolved flame front, with the propagation speed in the coarse cases around a factor of five greater than in the well-resolved cases.

The stochastic field thicknesses shown in Fig. 4 indicate that, for the well resolved simulations, the average thickness of the stochastic field reaction fronts is on the order of the laminar flame thickness at low Karlovitz numbers and increases with Karlovitz number and filter length scale due to the increasing contribution of sub-filter scale diffusivity. A similar trend can be seen for the coarse simulations, however the presence of numerical thickening is evident from the large increase of the normalised reaction front thickness as the Karlovitz number tends to zero. The effect of the conventional approach of setting Δ_*x*_ = Δ is to numerically thicken the reaction fronts so that on average they extend across at least twice the grid spacing, as shown by the Δ/*δ*_*L*_ = 1 results.

**Fig. 5:**
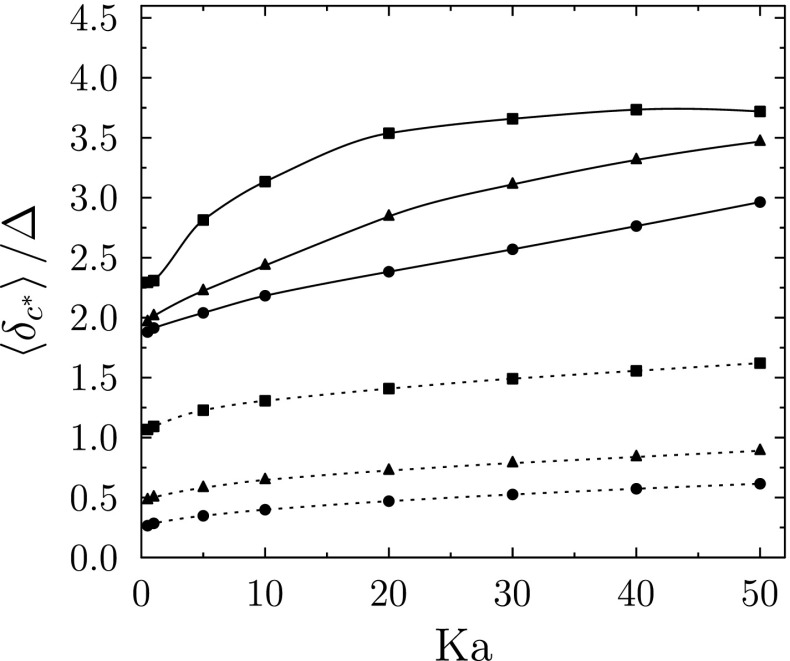
One-dimensional LES Stochastic Fields results for freely-propagating turbulent flame. Normalised resolved flame front speed (left) and average stochastic field thickness normalised by filter width (right) versus *Ka* for Δ/*δ*_*L*_ = 1, 2.5, 5. Refer to Fig. 4 for legend

An alternative illustration of the effect of numerical grid spacing on the mean stochastic field thickness is shown in Fig. 5 where the mean stochastic field thickness is normalised by the filter width used in the corresponding simulation. Given that an absolute minimum of five grid points are needed within a reaction front ($\delta _{c^{*}}/{\Delta }_{x}\ge 5$) in order to give acceptable numerical resolution, it is evident that the setting Δ_*x*_ = Δ does not satisfy this criterion anywhere in the wide range of combustion conditions considered.

The numerical thickening of reaction fronts is an error that changes the physical predictions of a three-dimensional LES in two main ways. First, the broadening of scalar gradients is expected to affect the local propagation speed of the reaction fronts in the Stochastic Fields. Second, the numerically-thickened reaction fronts are less-susceptible to wrinkling by the resolved turbulence leading to an under-prediction in the overall flame surface area.

The effect of the numerical thickening on the under-prediction of flame surface area and consumption rate cannot be evaluated directly from the one-dimensional simulations. Instead, we estimate the effect using established models for sub-filter scale flame wrinkling in the context of artificially-thickened flame modelling [16, 29]. The efficiency function developed by Charlette et al. [16] provides an estimate for the contribution to flame surface area from turbulent motions below a particular length scale. Evaluating the efficiency function for the reaction front thicknesses of the coarse and well-resolved simulations respectively, and taking their ratio,

11 $$ {\Theta}=\frac{\text{Efficiency function at coarse resolution}}{\text{Efficiency function at fine resolution}}, $$

gives an estimate for the amount of LES flame wrinkling that is lost due to numerical thickening of the reaction fronts.

The impact of the loss of flame surface area on burning rate due to numerical thickening is illustrated in Fig. 4 by multiplying the propagation speed data for Δ_*x*_ = Δ by Θ. For clarity within Fig. 4, the adjusted flame speeds are only shown for the Δ/*δ*_*L*_ = 1 and 5 cases. The magnitude of the adjustment to the coarse flame propagation data increases with increasing filter width due to the increase in numerical thickening associated with larger filter widths for a given Karlovitz number. It can be seen that even for a filter width of Δ/*δ*_*L*_ = 5, which is common in current laboratory scale flame simulations, around a 30% loss of turbulent flame area can be attributed to the numerical thickening due to use of the coarse grid. The projected 30% reduction in resolved flame surface area due to the use of coarse grid to some extent off-sets the erroneous five-fold increase in front propagation speed caused by the use of the coarse grid, but the combined effect is a significant net over-prediction of the resolved flame front speed, across the wide range of conditions investigated in this one-dimensional study. The impacts of these numerical errors have not been evaluated previously in relation to three-dimensional Stochastic Fields LES.

### Turbulent Bunsen flame LES

The F3 turbulent Bunsen flame of Chen et al. [15] is simulated by Stochastic Fields LES with coarse (Δ_*x*_ = Δ = 1.0 mm), fine (Δ_*x*_ = Δ = 0.5 mm) and improved (Δ_*x*_ = 0.5 mm, Δ = 1.0 mm) resolutions. The resolution of Δ_*x*_/*δ*_*L*_ = 1 is much finer than can be achieved in simulations of industrial high-pressure combustion systems, however the previous analysis of one-dimensional simulations indicates that even these grid spacings are inadequate to fully-resolve the Stochastic Fields solution.

**Fig. 6:**
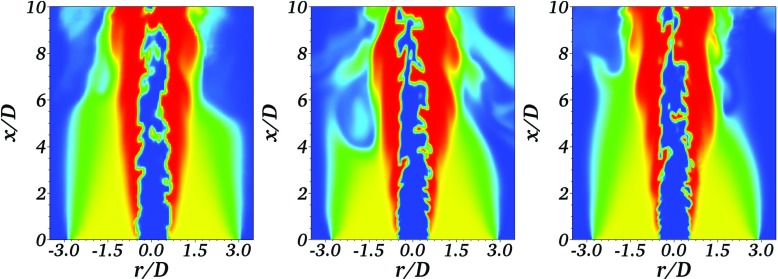
Instantaneous temperature field of an individual field from the turbulent Bunsen flame LES with Δ_*x*_ = Δ = 1.0 mm (left), Δ_*x*_ = 0.5 mm & Δ = 1.0 mm (mid), and Δ_*x*_ = Δ = 0.5 mm (right)

Figure 6 presents the instantaneous filtered temperature fields for an individual stochastic field in each of the three simulations. The coarse simulation exhibits less fine-scale structures than the other simulations. The temperature field in the improved resolution and fine simulations on the other hand show arguably similar levels of wrinkling. Given that the LES filter widths between the coarse and improved resolution are the same, the differences in wrinkling indicate that the reduction of visible flame wrinkling predominantly is due to numerical diffusion and thickening of the instantaneous field reaction front. The effect of numerical thickening is demonstrated by evaluating an indicative stochastic fields reaction front thickness given by the inverse average progress variable gradient magnitude on the Stochastic Fields conditioned on the progress variable giving maximum heat release, 〈|∇*ζ*_*c*,(*i*)_|∣*ζ*_*c*,(*i*)_ = 0.68〉^− 1^. The reaction front thicknesses of the Stochastic Fields for the coarse, improved resolution, and fine simulations are 2.68 mm, and 1.8 mm, and 1.71 mm respectively; demonstrating that the stochastic field thickness is still dependent on the numerical resolution, even with Δ_*x*_ = Δ/2.

**Fig. 7:**
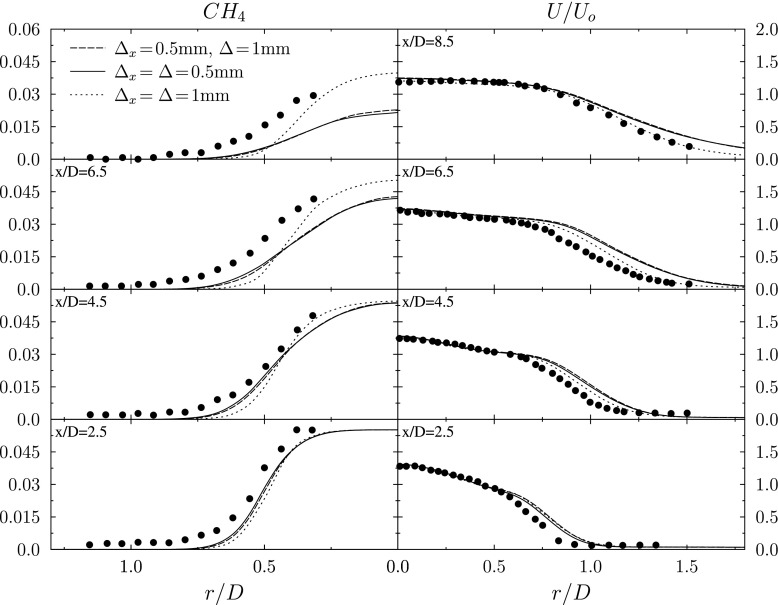
Radial distributions of the time-averaged methane mass fraction $\langle \widetilde {Y_{CH_{4}}}\rangle $ and normalised axial velocity at various axial locations

The radial distributions of normalised axial velocity are presented in Fig. 7 and are in reasonable agreement with the experimental measurements for all three simulations. The jet width predicted by the simulations is consistently greater than in the experiment, and this may be due to thermal expansion caused by the jet fluid at each axial location being more reacted in the simulation predictions than in the experimental measurements, as indicated by the methane mass fraction profiles also shown in Fig. 7. The similarity of the velocity field between the simulations and the experiment indicates that the average flow field is not strongly affected by the use of the different grids examined, and that the different LES provide similarly accurate flow fields in which to examine the effects of the Stochastic Field resolution.

The radial variation of the time-averaged filtered methane mass fraction and normalised axial velocity are shown in Fig. 7. The flame length is under-predicted in all of the simulations, with the coarse solution giving a longer flame length than the fine and improved resolution cases. The under-prediction of flame length corresponds to an over-prediction of propagation speeds of the LES-resolved flame fronts. The over-prediction of the propagation speed may be attributed to: (i) an increase in propagation speed due to under-resolution of the stochastic field reaction fronts, as anticipated by the preceding analysis of the one-dimensional flame simulations; (ii) the approximations used in modelling of the micromixing, spatial flux and reaction rate terms in the Stochastic Fields equations; and (iii) uncertainties in the inlet boundary conditions adopted for velocities and scalar variables.

In addition to the well-known phenomenon of numerical diffusion caused by under-resolution leading to enhanced propagation speeds, as outlined in previous sections, the increased numerical diffusion artificially thickens the flame and reduces its ability to be wrinkled by resolved scale turbulence. The longer flame height of the coarse simulation corresponds to a slower turbulent flame speed. The overall reduction in flame speed seen in the coarse case suggests that, for the particular case of the present Bunsen flame LES set up, the reduction in flame speed due to loss of flame surface area outweighs the flame speed enhancement associated with numerical diffusion within the flame front.

## Conclusions

The Stochastic Fields formulation is an attractive way to apply the transported probability density function approach to turbulent reacting flows, however the spatial resolution requirements become very demanding when applied to turbulent premixed combustion. Analysis of resolution requirements in one-dimensional Stochastic Fields simulations of premixed turbulent combustion shows that the thickness of the reaction fronts arising in the Stochastic Fields solution is on the order of the laminar flame thickness as the Karlovitz number tends to zero. In Large Eddy Simulation where the filter length scale can be much greater than the laminar flame thickness, the common practise of setting the LES filter scale equal to the grid spacing leads to very significant numerical thickening of the reaction fronts. The numerical diffusion caused by under-resolution affects the predicted turbulent flame speed in two ways: First, numerical diffusion increases the local propagation speed of the reaction fronts in the Stochastic Fields. Second, the numerically-thickened caused by the numerical diffusion makes the reaction fronts less susceptible to wrinkling by the resolved turbulence, leading to an under-prediction in the overall flame surface area. In combination, these two effects can have a leading-order effect on the predicted turbulent flame speed. This error is expected to be very significant in the simulation of high-pressure industrial combustion systems in which the grid spacing and filter length scales are typically an order of magnitude greater than the laminar flame thickness.

Three-dimensional Large Eddy Simulations of a laboratory turbulent Bunsen flame confirm that the Stochastic Fields solution is not numerically accurate when the grid spacing is equal to the filter length scale, whereas the flow field solution is relatively insensitive to further refinement of the spatial resolution. Even in relatively well-resolved LES (with filter scale equal to twice the laminar flame thickness), the impact of numerical inaccuracy on the overall burning rate is substantial. The present results provide a guide to the resolution required in order to obtain numerically-accurate Stochastic Fields solutions. The general requirement is that the grid spacing should be finer than the filter scale and the same order of magnitude as the laminar flame thickness, but this is impractical for many industrial applications involving high pressure combustion. In order to reduce the computational resolution requirements while retaining the key advantages of the Stochastic Fields method, a Thickened Stochastic Fields approach is developed in an accompanying paper [30]. Techniques such as level-set methods, or a form of adaptive grid refinement that ensures resolution of the individual stochastic fields could be considered in order to reduce the computational expense of solving the Stochastic Fields equations.
